# Ethnic Variations in Psychosocial and Health Correlates of Eating Disorders

**DOI:** 10.3390/healthcare6020038

**Published:** 2018-04-25

**Authors:** Shervin Assari, Mariana R. DeFreitas

**Affiliations:** 1 Department of Psychiatry, School of Medicine, University of Michigan, Ann Arbor, MI 48109-2029, USA; 2 Center for Research on Ethnicity, Culture and Health, School of Public Health, University of Michigan, Ann Arbor, MI 48109-2029, USA; 3 Health Behavior and Health Education, School of Public Health, University of Michigan, 1415 Washington Heights, Ann Arbor, MI 48109-2029, USA; 4 Medical School, University of Michigan Health System, Ann Arbor, MI 48109-2029, USA; mariana.r.defreitas@gmail.com

**Keywords:** ethnic groups, psychosocial factors, self-rated health, eating disorders, depression

## Abstract

The aim of this study is to explore ethnic variations in psychosocial and health correlates of eating disorders in the United States, Specifically, we compared associations between gender, socioeconomic status (SES), body mass index (BMI), physical and mental self-rated health (SRH), and major depressive disorder (MDD) with eating disorders (EDs) across 10 different ethnic groups in the United States. Data was obtained from the Collaborative Psychiatric Epidemiology Surveys (CPES), a national household probability sample collected in 2001–2003. Data for this study included a sample of 17,729 individuals with the following ethnic profile: 520 Vietnamese, 508 Filipino, 600 Chinese, 656 Other Asian, 577 Cuban, 495 Puerto Rican, 1442 Mexican, 1106 Other Hispanic, 4746 African American, and 7587 Non-Latino Whites. Gender, SES (education and income), BMI, SRH, MDD, and presence of EDs were measured across different ethnic groups. Logistic regression analysis was conducted for each ethnic group with lifetime EDs as the main outcome. Ethnic group varied in psychosocial and health correlates of EDs. In most ethnic groups, gender and SES were not associated with EDs. In almost all ethnic groups, EDs were associated with MDD and BMI. EDs were found to be associated with SRH in half of the ethnic groups studied. The associations between gender, SES, BMI, SRH, MDD, and EDs vary across different ethnic groups. These differences must be considered in further studies and in clinical practice in order to improve our approach towards diagnosis and treatment of EDs.

## 1. Introduction

Eating disorders (EDs)—such as binge eating disorder, anorexia nervosa, and bulimia nervosa—are associated with significant morbidity and mortality [1,2,3,4,5,6,7,8,9,10]. Comorbidities include liver dysfunction, electrolyte imbalances, osteoporosis, obesity, and heart disease [1,2,3,4]. In addition to medical comorbidities, EDs have been associated with psychiatric conditions such as major depressive disorder (MDD), general anxiety disorder (GAD), and obsessive compulsive disorders [5,6,7]. There is also evidence that individuals with EDs have significant increased risk in mortality [8,9,10]. Based on the National Comorbidity Survey Replication (NCS-R), the overall prevalence of EDs in the United States is between 3% and 6% [11]. There is a large body of studies examining the epidemiology of EDs including associations with other factors such as gender, socioeconomic status (SES), body mass-index (BMI), self-rated health (SRH), and MDD [12,13,14,15,16,17,18]. One of the shortcomings of most of these studies of ED epidemiology is the paucity of data on different races and ethnicities. As an example, 73% of NCS-R participants were Non-Hispanic Whites [19]. Given the serious implication of ED, a more thorough understanding of ED epidemiology in different ethnic groups is necessary. Amongst studies that have analyzed prevalence of EDs amongst different races and ethnicities, there have been some consistent findings such as increased ED prevalence in Asians [17,20,21] and decreased prevalence in African Americans [17,21,22]. There is limited data on the associations between ED prevalence and gender, SES, BMI, SRH, and MDD in the context of different ethnicities. A better understanding of these association has the potential of improving the outcomes of ED diagnosis and treatment in various populations.

There is a large body of studies showing an increased prevalence of EDs in women versus men [11,13,14]. However, there is also literature suggesting that are no significant differences in prevalence of EDs between women and men [23]. Therefore, current studies lack consistent findings. Additionally, a limitation of these studies is that the population considered was mostly Non-Hispanic White, ranging from 72% in Hudson et al. [11] to 89% in Lewinsohn et al. [15], resulting in a poor understanding of the prevalence of eating disorders in other races and ethnicities. Some of the existing literature has addressed gender differences in a single racial or ethnic group other than Non-Hispanic Whites. For example, in a study comparing gender differences amongst African Americans, no significant gender differences in overall prevalence of EDs were found [24]. Similarly, no significant differences in overall prevalence of EDs were found amongst Latinos [25]. Within the Asian population, women have exhibited a significantly higher prevalence of eating disorders compared to men [26]. Based on these studies, there may exist differences in gender prevalence amongst different ethnic groups. In our study, we will aim to look at these differences.

Examining the effects of SES on EDs may also reveal differences amongst different ethnicities. Existing literature reports a variety of findings with regards to SES and eating disorders. Some studies have shown no significant association between socioeconomic factors and EDs [27,28]. In contrast, some studies found an inverse relationship between SES and EDs regardless of race and ethnicity [16,17]. Yet, another study found that higher SES was associated with increased disordered eating [18]. Given the unclear association of SES in eating disorder, we will also aim to look at differences in the effects of SES on EDs in the context of different ethnicities.

With respect to BMI, previous studies have shed light on significant differences amongst ethnic groups in the effect of BMI on EDs [29,30,31]. For example, one study [29] revealed that some groups, such as Non-Hispanic White males, are more tolerant of larger BMI and thus, less likely to engage in disordered eating behavior. The same study observed a reversed pattern in Asian women. Other studies [30,31], looking at women only, concluded that Whites and Asians presented with body image discrepancy and disordered eating at lower BMIs compared to African Americans and Hispanics. The discrepancy in these findings could be due to gender differences rather than ethnic differences. We will aim to look at differences in the relationship of BMI and ED prevalence in different ethnicities regardless of gender.

Ethnic differences have been found in the context of SRH and psychiatric disorders such as MDD and GAD. One study compared the effects of GAD and MDD on SRH in African Americans and Caribbean Blacks and significant differences were found [32]. This study supports the need for additional research of psychiatric disorders and SRH to include more ethnic groups. A different study [33] analyzed SRH differences amongst several ethnic groups while taking BMI into consideration. Significant differences were found, with higher BMI being associated with poor SRH in Non-Hispanic Whites and a reversed pattern for African Americans [33]. Additionally, many studies have found obesity, body satisfaction, and depression varies amongst ethnic groups [34,35,36,37,38]. These studies suggest that ethnic groups perceive health and body weight differently, which can be associated with the heterogeneity in the prevalence of EDs amongst these groups. Therefore, we will also aim to examine the effects of SRH and MDD on EDs amongst different ethnicities.

In summary, all of these studies suggest that ethnic differences may exist in the association between EDs and gender, BMI, psychiatric disorders, and SRH. It is unclear if ethnic differences amongst different ethnicities between SES and EDs. These findings are of relevance, because all of these factors have been found to be associated with EDs [2,6,7,12,29,38,39], and EDs have been shown to affect individuals of all races and ethnicities [12]. However, significant gaps persist in understanding the association between EDs and these factors amongst different ethnic groups [40,41]. Examining these relationships will provide insight for more individualized care in the diagnosis and treatment of EDs in different ethnic groups. 

To enhance the existing knowledge on ethnic health disparities in EDs, this study explored ethnic variations in psychosocial and health correlates of EDs in the United States. Specifically, this study compared 10 different ethnic groups in the United States for the associations between gender, SES, BMI, physical and mental SRH, and MDD with EDs.

## 2. Methods

### 2.1. Design and Setting

This study was an analysis of data from the Collaborative Psychiatric Epidemiology Surveys (CPES) [42], which was funded by the National Institute of Mental Health (NIMH) and conducted between February 2001 and November 2003. The CPES is a composite of the National Comorbidity Survey Replication (NCS-R), the National Study of American Life (NSAL), and the National Latino and Asian American Study of Mental Health (NLAAS). Each of these surveys have employed similar methods, and collectively provide national data with sufficient power to examine racial and ethnical influences in mental disorders. Data was obtained by the Institute of Social Research (ISR), University of Michigan, Ann Arbor. Study design and sampling for CPES have been previously described [43]. 

### 2.2. Ethics

CPES participants provided written consent and received financial compensation for participation. The protocol was approved by the University of Michigan Review Board (IRB). Most interviews were face-to-face household surveys. Participants were provided financial incentive. The average response rate was 72.7% [42]. 

### 2.3. Sample

The participants were NCS-R (included a national household probability sample responders) (9282 individuals), NSAL responders (6082 individuals), and the NLAAS responders (4649 individuals). The study of 18,237 individuals with the following ethnic profile: 520 Vietnamese, 508 Filipino, 600 Chinese, 656 Other Asian, 577 Cuban, 495 Puerto Rican, 1442 Mexican, 1106 Other Hispanic, 4746 African American, and 7587 Non-Latino Whites. All participants were aged 18 or older. 

### 2.4. Measures

*Race and Ethnicity*. Race and ethnicity were self-identified in the CPES. The 10 racial and ethnic groups included (1) Vietnamese; (2) Filipino; (3) Chinese; (4) Other Asian; (5) Cuban; (6) Puerto Rican; (7) Mexican; (8) Other Hispanic; (9) African American; and (10) Non-Hispanic Whites. This variable was measured using multiple items on race, ethnicity, ancestry, and country of origin. 

*Gender*. Gender was a dichotomous measure and males were used as the reference category.

*Socioeconomic Factors*. The two measures used for socioeconomic determination were education level (categorical measure) and income (continuous measure). Education level categories were: less than high school (reference), high school graduate, some college, and college graduate.

*Body Mass Index (BMI)*. BMI was calculated based on self-reported weights in pounds and heights in feet and inches. Weights and heights were converted to metric system and BMI was obtained by calculating kg/m^2^. BMI was reported as a categorical measure as follows: underweight (BMI < 18.5 kg/m^2^), healthy weight (BMI = 18.5–24.9 kg/m^2^), overweight (BMI = 25.0–29.9 kg/m^2^), obese class I (BMI = 30.0–34.9 kg/m^2^), obese class II (BMI = 35–39.9 kg/m^2^), obese class III (BMI ≥ 40 kg/m^2^).

*Physical and Mental Self-Rated Health (SRH)*. This were assessed based on the answer to two questions: “How would you rate your overall physical?” and “How would you rate your overall mental health?” Both items had categorical measures with possible choices being: “excellent”, “very good”, “good”, “fair”, and “poor”. Responses were assigned numerical values with lower values corresponding to higher quality self-rated health. The validity of this two-item measure has been previously assessed and has been shown to be strongly associated with distress and well-being in racially/ethnically diverse populations [33,44,45]. 

*Major Depressive Disorder (MDD)*. The lifetime prevalence of depression was evaluated using the World Mental Health Composite International Diagnostic Interview (WMH-CIDI) [46], which is based on the Diagnostic and Statistical Manual of Mental Disorders, Fourth Edition (DSM-IV) and conducted by trained lay interviewers using probability samples. 

### 2.5. Statistical Analysis

#### 2.5.1. Sampling Weights

The CPES dataset includes sampling weights that are based on strata, clusters, and non-response. This is because the sampling strategy for all three sub-surveys in the CPES consisted of probability sampling proportional to size. As a result, there is a need to adjust for sampling weights, inversely proportional to these joint selection probabilities. Sampling weights are the reciprocals of the selection probabilities for the participant. Detailed descriptions of sample design and weighting procedures are available in a publication by Heeringa et al. (2004) [43].

#### 2.5.2. Analytical Approach

Given the complex sample design of CPES, Stata 13.0 (Stata Corp., College Station, TX, USA) was used for statistical analysis. Non-weighted and weighted frequencies for each ethnic group were reported. Mean and standard error of each study variable was reported for each ethnic group. All analysis used Taylor expansion technique for estimating standard error. Logistic regression was used for multivariable analysis by considering gender, SES, BMI, SRH, and MDD as predictors, and eating disorders as the outcome. Analysis was conducted separately for each ethnic group. Odds ratio, standard error, 95% confidence intervals, and *p*-values were reported. Statistical significance was defined by *p*-values of less than 0.05.

## 3. Results

### 3.1. Descriptive Statistics

Table 1 shows the sample sizes for each race and ethnic group in terms of percentage of responders (first column), number of responders (second column), and weighted sample size (third column). Based on weighted sample sizes, the responders are representative of over 200 million individuals in the United States.

Table 2 summarizes age, gender, years of education, income, eating disorder MDD, BMI classes, physical health ratings, and mental health ratings across different ethnic groups.

### 3.2. Associations of EDs

Table 3 summarizes the association of gender, SES, MDD, SRM, SRP, and BMI with the presence of EDs across 10 different ethnic groups.

## 4. Discussion

The goal of our study was to examine the association of gender, SES, BMI, SRH, MDD, with the presence of EDs across 10 different ethnic groups in the United States. For each ethnic group, there was at least one positive association between eating disorder prevalence and these factors. 

As suggested by prior literature [10,11,13], there is evidence for increased prevalence of EDs in women compared to men in the Non-Hispanic white populations. Interestingly, in our results, African Americans were the only other ethnic group where this positive association was also observed. No significant association was found between gender and ED prevalence in the other eight ethnic groups. Our study suggests that, as suspected, the association between gender and ED prevalence varies amongst different ethnic groups, and this must be considered in future studies of ED epidemiology. Additionally, providers must beware of clinical practice biases, such as higher index of suspicion of EDs in women, since many studies, including ours, have failed to find significant gender differences in certain ethnic groups. 

With regards to socioeconomic status, several ethnicities displayed a positive association between increasing level of education and income and prevalence of EDs, while others revealed no association. Our study did not find any support for an inverse relationship between ED prevalence and increased SES status in any of the ethnic groups studied. This heterogeneous pattern further supports the importance of awareness of these relationships in clinical practice and ethnic considerations in future studies of SES and ED epidemiology. Additionally, there is no substantial evidence of specific relationships between SES and EDs regardless of ethnicities. Therefore, it is crucial for providers to avoid interpreting SES as a risk factor for EDs.

For both mental self-rated health and physical self-rated health, a positive association was found between poor ratings and eating disorder prevalence in about half of the ethnic groups studied. In the Filipino, Chinese, and Puerto Rican populations no association was found between ED prevalence and either of these measures. The difference between these associations in various ethnic groups raises the possibility that cultural factors may play a role in how eating behaviors relate to an individual’s perception of both physical and mental health. Therefore, we advocate for cultural awareness to be a key component in the training of health care providers caring for individuals with EDs with the hopes that this will lead to more holistic and individualized treatment plans for these patients. 

Increasing BMI and presence of MDD displayed positive associations to EDs in almost all ethnic groups. This supports previous studies that have advocated for consideration of these factors in the context of ED diagnosis and treatment [15,31]. Exceptions in our studies were: no association between ED prevalence in both MDD and BMI in the Vietnamese and Chinese group, and no association between ED prevalence and MDD in Puerto Ricans. This suggests that, unlike the other factors, the consideration of ethnicity is less crucial when considering the association between these two factors and ED prevalence. Therefore, we recommend that future studies examining EDs in various ethnic groups be geared towards examining factors that have displayed greater heterogeneity in our studies than increasing BMI and presence of MDD. 

### 4.1. Limitations

There are a few limitations to this study. Firstly, the cross-sectional design of the study precludes any conclusions regarding causation. Secondly, the results are based on self-reported data. For example, BMI measures were based on self-reported weight and height. BMI has been found to have high correlations with measures of body fat [47,48], but the accuracy of self-reported data across various ethnic groups is unknown. Additionally, some categorical variables may have compromised the precision of the study. For example, the fact that education and BMI were listed as part of broad categories may mask some relationships. Also, since MDD and ED were binary variables, no conclusions of associations can be made based on severity of disease. Additionally, although single item-measure for SRH has established validity [33,44], it restricts response variability. Another limitation is the limited number of subgroups within major racial categories. For example, Asian subgroups represent a heterogeneous population, but only three distinct subgroups are represented in this study (Chinese, Vietnamese, and Filipino), with all other subgroups being aggregated in one category. This limits our understanding of eating disorder epidemiology within major racial groups. It is also important to consider that sample sizes differed amongst different ethnic groups, thus resulting in varying statistical power. Another limitation was a lack of data on interview modes, as people may differently respond to survey questions in face to face versus telephone surveys. Lastly, there may be limitations in validity of the survey across different ethnic groups; although there has been some work to ensure survey consistency across ethnic groups [49], unrecognized sources of bias may be at play. 

### 4.2. Future Directions

Our study revealed that there are considerable differences amongst ethnic groups in the associations between EDs and gender, SES, BMI, physical and mental SRH, and MDD. Additional studies are necessary to further analyze these differences, as the current literature on this topic is incomplete and has a variety of limitations. Particularly, the relationship of SRH and EDs in different ethnic groups could yield interesting findings, as a great level of heterogeneity was found in our study. Studying other factors such as body satisfaction and other psychiatric comorbidities in the context of different ethnicities will also provide further insight on our understanding of ED epidemiology. A better understanding of ED epidemiology will ultimately improve the diagnosis and treatment of the heterogeneous population of individuals suffering from EDs. Recognition of relevant subcultures. For example, African Americans are a complex group of multigenerational born, descendants of recent African Immigrants and Afro-Caribbeans and these differences show up in rates in depression which in this study is related to eating disorders. Moreover, there may be differences in types of eating disorders that would vary across ethnicity.

## 5. Conclusions

Demographic, social, and medical correlates of EDs vary across ethnic groups. Information regarding ethnic differences in correlates of EDs have clinical implication.

## Figures and Tables

**Table 1:** Weighted and unweighted sample size [40].

Ethnicity	%	*n*	Weighted N
Vietnamese	2.85	520	1,156,292
Filipino	2.79	508	1,909,580
Chinese	3.29	600	2,533,495
All Other Asian	3.60	656	3,452,027
Cuban	3.16	577	1,060,586
Puerto Rican	2.71	495	1,864,484
Mexican	7.91	1442	15,763,471
All Other Hispanic	6.06	1106	5,869,754
African American	26.02	4746	22,049,686
Non-Latino White	41.60	7587	148,000,000

**Table 2:** Descriptive statistics.

Variable	Vietnamese		Filipino		Chinese		All Other Asian		Cuban		Puerto Rican		Mexican		All Other Hispanic		African American		Non-Latino Whites	
	Mean	SE	Mean	SE	Mean	SE	Mean	SE	Mean	SE	Mean	SE	Mean	SE	Mean	SE	Mean	SE	Mean	SE
Age	43.73	0.67	42.98	0.75	42.88	0.61	38.10	0.68	48.97	0.73	41.17	0.72	36.68	0.48	38.38	0.52	42.19	0.27	46.73	0.45
Education	2.33	0.05	2.92	0.05	2.90	0.05	3.24	0.04	2.39	0.05	2.14	0.05	1.82	0.03	2.25	0.04	2.28	0.02	2.69	0.02
Household income	51.25	2.18	79.01	2.54	74.32	2.56	76.07	2.59	52.22	2.25	50.52	2.18	41.40	1.30	49.43	1.54	37.12	0.54	61.72	1.08
BMI classes	2.14	0.03	2.72	0.04	2.22	0.03	2.49	0.04	2.97	0.04	3.09	0.05	3.15	0.04	2.95	0.04	3.25	0.02	2.90	0.03
Mental self-rated health	2.40	0.05	2.00	0.04	2.41	0.04	1.86	0.04	2.17	0.05	2.22	0.05	2.32	0.03	2.11	0.03	2.15	0.02	2.18	0.02
Physical self-rated health	2.71	0.05	2.44	0.04	2.76	0.04	2.30	0.04	2.56	0.05	2.75	0.05	2.86	0.04	2.63	0.04	2.59	0.02	2.57	0.02

SE: Standard Error, BMI: Body Mass Index.

**Table 3:** Association of EDs with psychosocial and health factors in 10 ethnic groups.

Variable	Gender (Female)	Education	Income	MDD	Self-Rated Mental Health (Poor)	Self-Rated Physical Health (Poor)	BMI
Vietnamese	1.75 (0.21–14.50)	0.63 (0.34–1.17)	1.00 (1.00–1.00)	1.00 (1.00–1.00)	9.14 (2.04–40.84) **	6.23 (1.18–32.9) *	1.45 (0.65–3.24)
Filipino	1.53 (0.34–6.79)	0.57 (0.15–2.16)	1.00 (1.00–1.00) **	9.12 (1.94–42.84) **	1.00 (1.00–1.00)	3.2 (0.68–15.14)	1.65 (1.28–2.14) ***
Chinese	1.12 (0.15–8.57)	0.86 (0.36–2.03)	1.00 (1.00–1.00) ***	11.45 (0.87–150.35)	1.00 (1.00–1.00)	1.00 (1.00–1.00)	0.48 (0.09–2.46)
Other Asian	2.22 (0.41–12.06)	0.53 (0.17–1.65)	1.00 (1.00–1.00)*	12.05 (1.73–83.74) *	3.25 (0.24–44.50)	19.98 (2.65–150.54) **	2.55 (1.20–5.44) *
Cuban	2.58 (0.82–8.12)	0.88 (0.45–1.73)	1.00 (1.00–1.00)	6.6 (2.61–16.79) ***	3.32 (1.23–8.94) *	3.70 (1.07–12.87) *	1.47 (1.06–2.03) *
Puerto Rican	1.57 (0.40–6.11)	0.44 (0.21–0.93) *	1.00 (1.00–1.00)	2.41 (0.70–8.24)	2.86 (0.67–12.15)	3.27 (0.78–13.66)	2.07 (1.59–2.69) ***
Mexican	1.61 (0.71–3.67)	1.01 (0.75–1.36)	1.00 (1.00–1.00)	4.83 (2.29–10.19) ***	0.26 (0.05–1.42)	2.56 (1.38–4.74) **	1.76 (1.30–2.38) ***
Other Hispanic	1.04 (0.26–4.07)	1.00 (0.62–1.63)	1.00 (1.00–1.00)	3.77 (1.37–10.37) *	4.13 (1.35–12.63) *	1.91 (0.82–4.46)	1.46 (1.04–2.04) *
African American	2.50 (1.27–4.94) **	0.73 (0.56–0.96) *	1.00 (1.00–1.00)	4.62 (2.51–8.54) ***	4.13 (2.37–7.24) ***	3.61 (2.05–6.36) ***	1.59 (1.28–1.98) ***
Non-Hispanic Whites	2.17 (1.27–3.72) **	0.91 (0.63–1.31)	1.00 (1.00–1.00)	4.35 (2.27–8.36) ***	6.07 (1.59–23.22) *	1.77 (0.50–6.27)	1.51 (1.29–1.76) ***

MDD: Major Depressive Disorder (Lifetime), * *p*-value < 0.05, ** *p*-value < 0.01, *** *p*-value < 0.001.
